# N-glycosylation acts as a switch for FGFR1 trafficking between the plasma membrane and nuclear envelope

**DOI:** 10.1186/s12964-023-01203-3

**Published:** 2023-07-21

**Authors:** Paulina Gregorczyk, Natalia Porębska, Dominika Żukowska, Aleksandra Chorążewska, Aleksandra Gędaj, Agata Malinowska, Jacek Otlewski, Małgorzata Zakrzewska, Łukasz Opaliński

**Affiliations:** 1 Faculty of Biotechnology, Department of Protein Engineering, University of Wroclaw, Joliot-Curie 14a, 50-383 Wroclaw, Poland; 2 Institute of Biochemistry and Biophysics, Polish Academy of Sciences, Pawińskiego 5a, 02-106 Warsaw, Poland

**Keywords:** Glycosylation, FGFR1, Trafficking, Nucleus, Signaling

## Abstract

Fibroblast growth factor receptor 1 (FGFR1) is a heavily N-glycosylated cell surface receptor tyrosine kinase that transmits signals across the plasma membrane, in response to fibroblast growth factors (FGFs). Balanced FGF/FGFR1 signaling is crucial for the development and homeostasis of the human body, and aberrant FGFR1 is frequently observed in various cancers. In addition to its predominant localization to the plasma membrane, FGFR1 has also been detected inside cells, mainly in the nuclear lumen, where it modulates gene expression. However, the exact mechanism of FGFR1 nuclear transport is still unknown. In this study, we generated a glycosylation-free mutant of FGFR1, FGFR1.GF, and demonstrated that it is localized primarily to the nuclear envelope. We show that reintroducing N-glycans into the D3 domain cannot redirect FGFR1 to the plasma membrane or exclude the receptor from the nuclear envelope. Reestablishment of D2 domain N-glycans largely inhibits FGFR1 accumulation in the nuclear envelope, but the receptor continues to accumulate inside the cell, mainly in the ER. Only the simultaneous presence of N-glycans of the D2 and D3 domains of FGFR1 promotes efficient transport of FGFR1 to the plasma membrane. We demonstrate that while disturbed FGFR1 folding results in partial FGFR1 accumulation in the ER, impaired FGFR1 secretion drives FGFR1 trafficking to the nuclear envelope. Intracellular FGFR1.GF displays a high level of autoactivation, suggesting the presence of nuclear FGFR1 signaling, which is independent of FGF. Using mass spectrometry and proximity ligation assay, we identified novel binding partners of the nuclear envelope-localized FGFR1, providing insights into its cellular functions. Collectively, our data define N-glycosylation of FGFR1 as an important regulator of FGFR1 kinase activity and, most importantly, as a switchable signal for FGFR1 trafficking between the nuclear envelope and plasma membrane, which, due to spatial restrictions, shapes FGFR1 interactome and cellular function.

Video Abstract

**Supplementary Information:**

The online version contains supplementary material available at 10.1186/s12964-023-01203-3.

## Background

Fibroblast growth factors (FGFs) and fibroblast growth factor receptors (FGFRs) are signal transduction platforms controlling fundamental cellular processes such as differentiation, division, motility or death [1, 2]. FGFs/FGFRs are frequently altered in malignancies and constitute targets for selective cancer treatment [3–6]. The FGF family includes 22 proteins, most of which contain an N-terminal signal peptide (SP) that directs FGFs into the secretory pathway and ensures their extracellular localization [7]. Similarly, all four FGFRs contain SPs that, together with a single transmembrane region (TM), ensure the integration of FGFRs into the plasma membrane. Thus, canonical FGF/FGFR signaling units operate at the cell surface, where extracellular ligands, FGFs, recognize, dimerize and activate cognate receptors, FGFRs, and initiate intracellular signaling involving the phospholipase Cγ (PLCγ), signal transducer and activator of transcription (STAT), phosphoinositide 3-kinase (PI3K)/protein kinase B (AKT)/mammalian target of rapamycin (mTOR) and Ras–Raf–MEK–ERK pathways [2, 8, 9] In addition to signaling at the cell surface, FGFs and FGFRs reside inside the cell, mainly in the cytosol and nucleus [10]. For instance, the FGFs subfamily, fibroblast growth factor homologous factors (FHFs), are cytosolic regulators of plasma membrane ion channels and components of the nucleolar ribosome assembly complex [11, 12]. FGF1 and FGF2 are internalized via FGFR-mediated endocytosis, escape from endosomes and use their nuclear localization signals (NLS) for nuclear translocation [13]. Similarly, nuclear localization of FGFRs has been demonstrated in many tissues and tumors [1, 14–16]. FGFR1 is FGFR member that is the most commonly overexpressed in malignancies, particularly in breast cancer, small cell lung cancer, urothelial carcinoma and head and neck cancer [3]. FGFR1 consists of an extracellular region divided into three immunoglobulin-like domains (D1-D3), a TM region and an intracellular split tyrosine kinase domain [17]. Notably, the extracellular part of FGFR1 is highly N-glycosylated, as it contains eight N-X-S/T glycosylation sites distributed in the D1 (2 motifs), D2 (2 motifs) and D3 (4 motifs) domains [18]. N-glycosylation regulates the interaction of FGFR1 with FGFs, extracellular galectins and heparan-sulphate co-receptors [18, 19]. Although the main signaling activity of FGFR1 occurs at the cell surface, numerous studies reported FGFR1 inside the cell, mainly in the nucleus, where it interacts with several proteins, including CBP/CREB complex, Nurr1, RNA polymerase II or FOXA1, and regulates gene expression in a tyrosine kinase-independent manner [15, 16, 20–31]. Although several pathways for the nuclear translocation of FGFR1 have been proposed, the precise mechanism of FGFR1 accumulation in the nucleus is still unclear. In the first model of the FGFR1 nuclear transport pathway, the full-length N-glycosylated FGFR1 (FGF- stimulated or not) is internalized from the cell surface via clathrin-mediated endocytosis (CME) or clathrin-independent endocytosis (CIE) and a portion of the receptor retro-translocates through the ER to the nucleus in an importin-β-dependent manner, suggesting the involvement of the nuclear pore complex (NPC) in the nuclear import of FGFR1 [22, 30]. In the second mechanism, soluble FGFR1 is released into the cytosol during/after synthesis in the ER, where it associates with FGF2 and RSK1, which provide a nuclear localization signal (NLS) for import of FGFR1-containing complex into the nucleus in an importin-β-dependent manner [22, 32]. In the third scenario, limited proteolysis of FGFR1 by granzyme B generates a C-terminal intracellular soluble region of FGFR1 that is transported to the nucleus [33]. Interestingly, it was observed that hypo-glycosylated FGFR1 is preferentially localized to the nucleus in the bovine adrenal medullary cells (BAMC) cells, implicating that N-glycosylation of FGFR1 may play an important role in cellular trafficking of FGFR1 [34, 35]. Here, we have investigated the importance of N-glycosylation of FGFR1 for its cellular trafficking and provided solid evidence for the relocation of FGFR1 to the nuclear envelope, which is tightly dependent on the N-glycosylation status of the receptor. Furthermore, we have demonstrated that nuclear envelope-localized FGFR1 is activated in a ligand-independent manner and is engaged in a discrete set of interactions with nuclear proteins, providing insights into novel functions of nuclear FGFR1.

## Methods

### Antibodies and reagents

The primary antibodies directed against FGFR1 (#9740), phospho-FGFR (p-FGFR1, #3476) and lamin A/C (#4777) were obtained from Cell Signaling (Danvers, MA, USA). The antibodies directed against COPB (#sc-393615), FGF-2 (#sc-74412), heterogenous nuclear ribonucleoproteins C1/C2 (#sc-32308), HSP90 (#sc-13119), U4/U6.U5 tri-snRNP-associated protein 1 (#sc-376460), protein disulfide isomerase A4 (#sc-390530), nucleolin (#sc-8031), SBP-tag (#sc-101595) were purchased from Santa Cruz Biotechnology (Dallas, TX, USA). Anti-importin α-1 primary antibody (#DF6510) was from Affinity Bioscience (Melbourne, VIC, AU). For detection of FGFR1 in immunofluorescence microscopy experiments high affinity, highly selective tetravalent anti-FGFR1 T-Fc antibody was used [36]. HRP-conjugated secondary antibodies and secondary anti-mouse antibody conjugated to Alexa Fluor 594 (#715–585-150) were from Jackson Immuno-Research Laboratories (Cambridge, UK). The secondary anti-rabbit antibody conjugated to Alexa Fluor 594 (#A11037) and Zenon-AF-488 were from Thermo Fisher Scientific (Waltham, MA, USA). Streptavidin Agarose resin (#20,349) was from Thermo Fisher Scientific (Waltham, MA, USA). Heparin Sepharose 6 Fast Flow resin (#GE17-0998–01) and Ni Sepharose 6 Fast Flow resin (#GE17-5318–02) were from GE Healthcare (Chicago, IL, USA). Nonidet P-40 (#NON505) were from BioShop (Burlington, ON, CA). Protease Inhibitors Cocktail (# was from Roche Diagnostics GmbH (Indianapolis, IN, USA). Tunicamycin was from Santa Cruz Biotechnology, HA15, 17-AAG, PD173074 and brefeldin A were from Merck (Darmstadt, Germany). Recombinant FGF1 and FGF2 were obtained as described previously [37, 38].

### Cells

Human osteosarcoma cell line (U2OS) was obtained from American Type Culture Collection (ATCC, Manassas, VA, USA). U2OS cell lines stably transfected with pcDNA3.1 vector containing the sequence encoding SBP-FGFR1 (U2OS-SBP-R1) and SBP-FGFR1.GF (U2OS-SBP-R1.GF) were prepared as described previously [39]. Transient transfections of U2OS cells with pcDNA3.1 vectors containing sequences encoding FGFR1 and FGFR1.GF variants (Gene Universal, Newark, DE, USA) were performed with FuGENE® HD Transfection Reagent (Promega), according to the manufacturer’s instructions. Cells were cultivated in DMEM (Biowest, Nuaille, France) supplemented with 10% fetal bovine serum (Thermo Fisher Scientific, Waltham, MA, USA) and antibiotics (100 U/mL penicillin, 100 μg/mL streptomycin). For U2OS-SBP-R1 and U2OS-SBP-R1.GF cells growth media were additionally supplemented with geneticin (1.0 mg/mL and 1.5 mg/mL respectively) (Thermo Fisher Scientific, Waltham, MA, USA). All cells were cultivated in 5% CO2 atmosphere at 37 °C and seeded onto tissue culture plates one day prior start of the experiments.

### Mutagenesis

The pcDNA 3.1-based genetic constructs for expression of FGFR1.GF variants with reintroduced mutations to alanines were obtained via gene synthesis (Gene Universal, Newark, DE, USA) or prepared using site-directed mutagenesis using Phusion™ Site-Directed Mutagenesis Protocol (Thermo Fisher Scientific) with pcDNA 3.1—FGFR1.GF as a template. The correctness of all genetic constructs was confirmed by DNA sequencing.

### FGFR1 activation

Serum-starved U2OS cells and U2OS cells transfected with the vectors containing sequences encoding FGFR1 and FGFR1.GF (both stable and transient transfectants) were stimulated for 15 min with FGF1 (100 ng/mL). Cells were lysed in Laemmli buffer and subjected to SDS-PAGE and western blotting. Densitometric analyses of FGFR signaling were performed with ImageLab 5.0 software (Biorad). Average values of 3 independent experiments ± SD are shown. Statistical analyses were performed with Student’s t-test (*p < 0.05; **p < 0.005 and ***p < 0.001; n.s. – not significant).

### Affinity purification of SBP-FGFR1 GF complexes for mass spectrometry

U2OS-R1 cells (control, producing untagged FGFR1), U2OS-SBP-R1 cells (producing SBP-FGFR1) and U2OS-SBP-R1.GF cells (producing SBP-FGFR1.GF) (4 × 10^6^ for each cell line per isolation) were serum starved for 4 h. Cells were washed with PBS and lysed with Lysis Buffer (LB: 50 mM Tris, 150 mM NaCl, 1 mM EDTA, 0.1% Nonidet P-40, 1 mM PMSF, Protease Inhibitors Cocktail, pH 8.0). Lysate was briefly sonicated and subjected to clarifying spin (14,000 rpm, 10 min, 4 °C). Supernatant was incubated overnight at 4 °C with LB-equilibrated Streptavidin-Agarose resin with shaking. Beads were washed with Washing Buffer (WB: 50 mM Tris, 150 mM NaCl, 1 mM EDTA, pH 8.0) and with PBS. Beads containing bound proteins were subsequently subjected to label-free quantitative comparative mass spectrometry (MS) analyses.

### Mass spectrometry

#### Sample preparation and measurement

Mass spectrometry experiments were performed at the Mass Spectrometry Laboratory at the Institute of Biochemistry and Biophysics PAS. The submitted samples contained agarose beads with bound proteins. At first, cysteines were reduced by 1 h incubation with 20 mM tris(2-carboxyethyl) phosphine (TCEP) at 60 °C followed by 10 min incubation at a room temperature with 50 mM methyl methanethiosulfonate (MMTS). Digestion was provided at 37 °C overnight with 1 µg of trypsin (Promega). Tryptic digestion was stopped by lowering the pH of reaction below pH 4 by adding extraction buffer (0.1% TFA 2% acetonitrile). Agarose beads were separated from solution by centrifuging. Samples were analysed using LC–MS system composed of Evosep One HPLC System (Evosep Biosystems) coupled to an Orbitrap Exploris 480 mass spectrometer (Thermo Scientific). 20 µl of peptide solution (1/4 of sample) was loaded onto Evotips C18 disposable trap column as described [40]. Peptides were fractionated using 88 min (15 samples per day) predefined Evosep gradient at a flow rate of 250 nl/min on an analytical column (Dr Maisch C18 AQ, 1.9 µm beads, 150 µm ID, 15 cm long, Evosep Biosystems). Data-dependent acquisition parameters were as follows: top 25 precursors selected for MS2 analysis, collisional induced fragmentation NCE 30%, spray voltage 2.1 kV, funnel RF level 40, heated capillary temperature to 275 °C. Full MS scans covering the mass range of 300–1700 m/z with a resolution of 60,000, a maximum injection time set to Auto and a normalized AGC target to 300%. MS2 scans were acquired with a resolution of 30,000, an Auto maximum injection time and a Standard AGC target. Ion isolation window was set to 1.2 m/z with Precursor Fit at 70%, a dynamic exclusion to 20 s and a minimum intensity threshold at 5e3.

#### Data analysis

The acquired MS/MS data were pre-processed with Mascot Distiller software (v. 2.8, MatrixScience, London, UK) and a search was performed with the Mascot Search Engine (MatrixScience, London, UK, Mascot Server 2.8) using database of human proteins derived from Swissprot (20,436 sequences) supplemented with popular MS contaminants. To reduce mass errors, the peptide and fragment mass tolerance settings were established separately for individual LC–MS/MS runs after a measured mass recalibration – typical tolerance value for parent ions was 5 ppm and for fragment ions – 0.01 Da. The rest of search parameters were as follows: enzyme, Trypsin; missed cleavages, 1; fixed modifications, Methylthio (C); variable modifications, Oxidation (M); instrument, HCD. A statistical assessment of the confidence of peptide assignments was based on the target/decoy database search strategy. Proteins identified by a subset of peptides were removed from analysis. Proteins that exactly matched the same set of peptides were combined into a single group (family). The mass calibration and data filtering described above were carried out with MScan software, developed in-house (http://proteom.ibb.waw.pl/mscan/).

The lists of identified peptides were merged into one common list. This list was overlayed onto 2-D heatmaps generated from LC–MS/MS datasets by tagging the peptide-related isotopic envelopes with corresponding peptide sequence tags on the basis of the measured/theoretical mass difference, the deviation from the predicted elution time and the match between theoretical and observed isotopic envelopes. A more detailed description of the quantitative extraction procedure implemented by our in-house software is available in [41]. The abundance of each peptide was determined as the height of a 2-D fit to the monoisotopic peak of the tagged isotopic envelope. Quantitative values were next exported into text files, along with peptide/protein identifications, for statistical analysis with Diffprot software [42]. Diffprot was run with the following parameters: number of random peptide sets = 106; clustering of peptide sets – only when 90% identical; normalization by LOWESS.

The mass spectrometry proteomics data have been deposited to the ProteomeXchange Consortium via the PRIDE partner repository with the dataset identifier PXD042325 and 10.6019/PXD042325 [43].

### Pull down

To study the interaction of FGF2 with FGFR1, U2OS-SBP-R1 and U2OS-SBP-R1.GF cells (2 × 10^6^ for each cell line per isolation) were washed with PBS and lysed with Lysis Buffer (LB: 50 mM Tris, 150 mM NaCl, 1 mM EDTA, 0.1% Nonidet P-40, 1 mM PMSF, Protease Inhibitors Cocktail, pH 8.0). Lysate was briefly sonicated and subjected to clarifying spin (14,000 rpm, 10 min, 4 °C). Supernatant was incubated for 1 h at 4 °C with LB-equilibrated Streptavidin-Agarose resin with shaking. Beads were washed with LB and PBS. Next, FGF2 was added (5 μg/sample) and incubated at the same conditions. Beads were subsequently washed with LB containing 0,3% Nonidet P-40 and with PBS. As a control for unspecific FGF2 interaction with the beads, a resin sample without immobilized lysate was used. Proteins were eluted with Laemmli buffer, separated using SDS-PAGE and analyzed with western blotting using antibodies recognizing FGF2 and FGFR1.

### Proximity ligation assay (PLA)

To analyze the interactions between FGFR1.GF, FGFR1 and their protein partners, Duolink® In Situ Fluorescence Protocol was used (Sigma-Aldrich). U2OS-SBP-R1.GF cells were fixed with 4% paraformaldehyde and permeabilized with 0.1% Triton in PBS. Cells were then incubated with appropriate antibodies and treated according to the manufacturer’s protocols. Cell nuclei were stained with NucBlue Live dye.

### Fluorescence microscopy

For the analysis of FGFR1 variants’ subcellular localization, cells were washed with PBS, fixed with 4% paraformaldehyde and permeabilized with 0.1% Triton in PBS. Next, cells were incubated with anti-FGFR1 recombinant antibody T-Fc at 37 °C for 30 min. T-Fc was visualized with Zenon AF-488 (Thermo Fisher Scientific) and nuclei were labeled with a NucBlue Live dye (Thermo Fisher Scientific). The subcellular localization of FGFR1 in U2OS-R1 cells upon treatment with tunicamycin (0.5 μg/mL, 24 h), HA15 (10 μM, 24 h), 17-AAG (100 nM, 24 h) and brefeldin A (2.5 μM, 2 h) was studied as described above. The specificity of the anti-FGFR1 antibody was verified in parental FGFR1-negative U2OS cells. The intracellular co-localization of FGFR1.GF with ER and nuclear envelope markers was analyzed with immunofluorescence using anti-COPB and anti-lamin A/C antibodies, as described previously [44]. The co-localization of auto-activable pool of FGFR1.GF with the nuclear envelope was performed using anti-phospho-FGFR antibodies (pFGFR). The specificity of pFGFR antibodies was verified with western blotting and immunofluorescence in U2OS-R1 cells pretreated with PD173074 (100 nM for 15 min). FGF1 was fluorescently labelled with DyLight550 and its internalization was studied as described previously [37]. As secondary antibodies, anti-rabbit and anti-mouse antibodies conjugated to Alexa Fluor 594 (Thermo Fisher Scientific) were used. Wide field fluorescence microscopy was carried out using a Zeiss Axio Observer Z1 fluorescence microscope (Zeiss, Oberkochen, Germany) as described previously [45]. Image analysis was carried out using Zeiss ZEN 2.3 software (Zeiss, Oberkochen, Germany) and Adobe Photoshop (Adobe, San Jose, CA, USA).

## Results

### Glycosylation-deficient mutant of FGFR1 displays high level of autoactivation

**Fig. 1:**
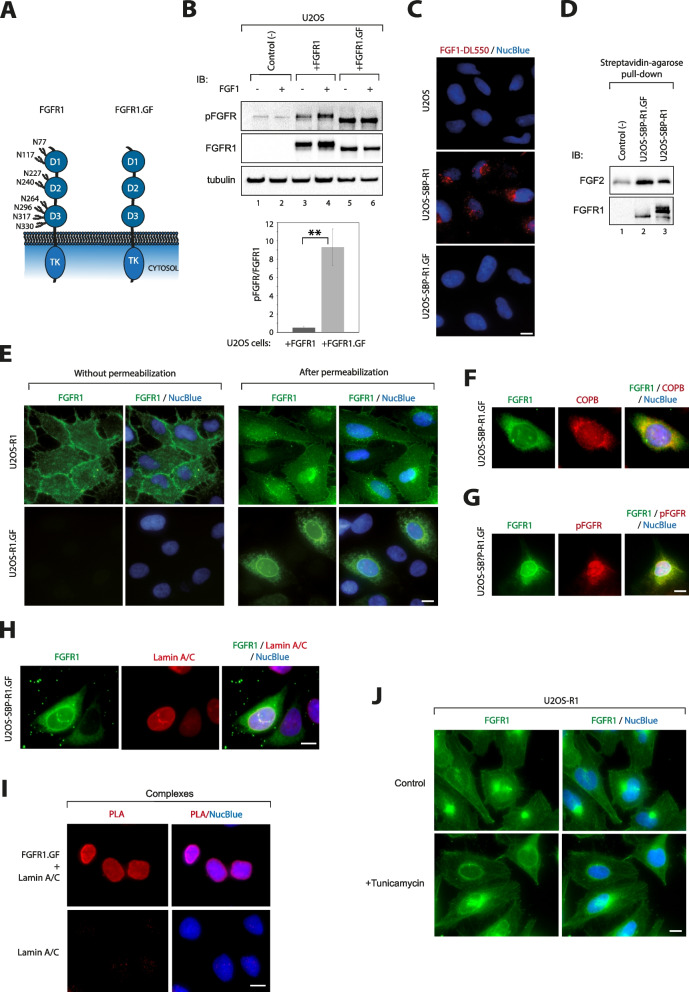
Glycosylation-deficient FGFR1 is localized to the nuclear envelope where it displays high level of autoactivation. **A**. Schematic representation of the structure of FGFR1 and FGFR1.GF. Positions of the N-glycosylation sites within the extracellular domain of the receptor are marked. **B**. FGFR1 activation in the presence or absence of FGF1 (100 ng/mL) in U2OS cells (control) and U2OS cells transfected with FGFR1 and FGFR1.GF. Tubulin served as a loading control. Quantification of pFGFR/FGFR1 signals was performed with ImageLab 5.0 software. Average values of 3 independent experiments (*n* = 3) ± SD are shown. Statistical analyses were performed with Student’s t-test (*p < 0.05; **p < 0.005 and ***p < 0.001; n.s. – not significant). **C**. Internalization of fluorescently labeled FGF1 into U2OS, U2OS-R1 and U2OS-R1.GF cells. Cells were incubated with FGF1-DL550 (500 ng/mL) and heparin (10 U/uL) for 30 min at 37 °C, cells were fixed, nuclei were stained with NucBlue dye and cells were analyzed by fluorescence microscopy (*n* = 3). Scale bar represents 10 µm. **D**. Pull down experiment demonstrating interaction between FGF2 and SBP-FGFR1 or SBP-FGFR1.GF. U2OS cells stably producing receptor variants were lysed, receptor was captured on streptavidin-agarose beads and beads were then incubated with recombinant FGF2. After extensive washing, co-purification of FGF2 with SBP-FGFR1 and SBP-FGFR1.GF was analyzed by western blotting. Empty beads served as a control for specificity of FGF2 purification with FGFR1 variants (*n* = 3). **E**. Subcellular localization of FGFR1 and FGFR1.GF. FGFR1 variants in transiently transfected U2OS cells were detected by immunofluorescence in permeabilized and non-permeabilized cells. Nuclei were labelled with NucBlue dye (*n* = 3). Scale bar represents 10 µm. **F**. Immunofluorescence-based co-localization of SBP-FGFR1.GF with the ER/Golgi marker protein COPB (*n* = 3). Scale bar represents 20 µm. **G**. Immunofluorescence-based co-localization of SBP-FGFR1.GF with pFGFR signal (*n* = 3). Scale bar represents 20 µm. **H**. Immunofluorescence-based co-localization of SBP-FGFR1.GF with the nuclear envelope marker proteins lamins A/C (*n* = 3). Scale bar represents 20 µm. **I**. PLA-based analysis of SBP-FGFR1.GF interaction with lamins A/C in the nuclear envelope (*n* = 3). Scale bar represents 20 µm. **J**. Localization of FGFR1 in U2OS-R1 cells (control) and in U2OS-R1 cells treated with tunicamycin (*n* = 3). Scale bar represents 20 µm

To study the involvement of N-glycosylation in the function and cellular trafficking of FGFR1, we generated an expression vector allowing for production of glycosylation-free variant of the receptor (FGFR1.GF) by mutating all eight N-S–S/T motifs present in its extracellular region (Fig. 1A). We transfected U2OS cells with wild type FGFR1 or FGFR1.GF and used western blotting to monitor the glycosylation status of FGFR1. In contrast to wild type FGFR1, detected as several bands representing distinct N-glycosylation species of the receptor, FGFR1.GF migrated as a single band of lower molecular weight, indicating an effective removal of N-glycans from the receptor by introduced mutations (Fig. 1B, lanes 3 and 5). Interestingly, we detected much higher level of FGFR1.GF phosphorylation without ligand stimulation compared to wild type FGFR1 (Fig. 1B, lanes 3 and 5, and graph). We observed that serum-starved U2OS-R1.GF cells did not respond to FGF1 treatment, while we detected an increased phosphorylation of wild type FGFR1 produced by U2OS cells (Fig. 1B, lanes 4 and 6). We tagged N-terminally wild type FGFR1 and FGFR1.GF with streptavidin binding peptide (SBP) and prepared stable transfectants of U2OS cells with these receptor variants (Figure S1). Using stably transfected U2OS-SBP-R1.GF cells, we confirmed their higher level of pFGFR/FGFR1 ratio in relation to the control U2OS-SBP-R1 cell line and the lack of response of U2OS-SBP-R1.GF to FGF1 stimulation (Figure S1).

Following FGF1 interaction with FGFR1 and subsequent receptor dimerization, the FGF1/FGFR1 complex is internalized by cells mainly through clathrin-mediated endocytosis [46–53]. We assessed the cellular uptake of fluorescently labelled FGF1 by U2OS cells (lacking detectable levels of FGFR1), U2OS-SBP-R1 and U2OS-SBP-R1.GF using fluorescence microscopy. As expected, we observed numerous punctate FGF1 signals inside U2OS-R1 cells and virtually no signal for U2OS cells (Fig. 1C). Interestingly, for U2OS-R1.GF, FGF1 signal was barely detectable, similarly to that for U2OS cells (Fig. 1C).

These data indicate that the glycosylation-deficient mutant of FGFR1 is properly folded at least in the intracellular domain, as it is kinase-active. FGFR1.GF shows significantly increased autoactivation relative to the wild-type receptor, suggesting that N-glycosylation of FGFR1 may prevent receptor autoactivation in the absence of ligands. Furthermore, our results indicate that FGFR1.GF is unable to respond to FGF1 stimulation.

### Glycosylation-deficient FGFR1 is predominantly localized to the nuclear envelope

The lack of response of FGFR1.GF to FGF1 stimulation could result from its inability to interact with the ligand or from a spatial restriction for FGF1/FGFR1.GF complex assembly caused by FGFR1.GF mislocalization. To test the first possibility, we assessed the interaction of FGFR1.GF with recombinant FGF2 (as FGF1 displayed strong binding to streptavidin agarose beads) by pull down. We isolated SBP-FGFR1 and SBP-FGFR1.GF from the corresponding stably transfected cell lines using streptavidin-agarose beads, and receptor variants were incubated with recombinant FGF2. Formation of receptor-ligand complexes was assessed with western blotting. As shown in Fig. 1D, FGF2 co-purified with SBP-FGFR1 and SBP-FGFR1.GF with similar efficiency, indicating that FGFR1.GF is capable of binding FGF2.

Next, to investigate whether mislocalization of FGFR1.GF might stand behind the lack of receptor response to FGF1, we performed immunofluorescence microscopy. Subcellular localization of FGFR1 and FGFR1.GF was assessed in U2OS-R1 and U2OS-R1.GF cells using highly specific, high affinity recombinant anti-FGFR1 antibody T-Fc (Figure S2A) [36]. Incubation of cells with the anti-FGFR1 antibody without cell permeabilization under conditions that impede endocytosis (4°C) resulted in a clearcut cell-surface staining in U2OS-R1 cells and a complete absence of signal for U2OS-R1.GF (Fig. 1E). Interestingly, when permeabilized U2OS-R1.GF cells were incubated with T-Fc, a predominant FGFR1.GF signal encircling the nuclei-specific dye NucBlue was observed, indicating the prevalent localization of FGFR1.GF to the nuclear envelope (Fig. 1E). Besides the nuclear envelope, we detected FGFR1.GF in additional proximal structures of the nucleus, which largely co-localized with the ER/Golgi marker COPB (Fig. 1F). In contrast, wild type FGFR1 signal in permeabilized cells was detected at the cell surface and in intracellular spots probably representing endosomes or secretory vesicles (Fig. 1E). To study whether the highly auto-activable FGFR1.GF pool (Fig. 1B) includes FGFR1.GF localized to the nuclear envelope, we performed immunofluorescence microscopy using anti-phospho-FGFR (pFGFR) antibodies, for which we verified specificity with the FGFR kinase inhibitor PD173074 (Fig. S2B). As expected, tyrosine-phosphorylated FGFR1.GF accumulated in the nuclear envelope (Fig. 1G). Additionally, we assessed the co-localization of FGFR1.GF with lamins A/C, nuclear envelope marker proteins. As shown in Fig. 1H, co-localization of the nuclear ring signal of FGFR1.GF with that of lamins A/C was detected. To further confirm that FGFR1.GF is present in the nuclear envelope, we studied FGFR1.GF interaction with lamins A/C using a proximity ligation assay (PLA). A clear PLA signal concentrating at the nuclear ring was detected for FGFR1.GF – lamins A/C (Fig. 1I).

To additionally study whether the trafficking of FGFR1.GF to the nuclear envelope is triggered by the lack of receptor N-glycosylation, we chemically blocked N-glycosylation in U2OS-R1 cells with tunicamycin and studied the subcellular localization of FGFR1 with fluorescence microscopy. In agreement with the results for FGFR1.GF, chemical inhibition of cellular N-glycosylation resulted in diminished cell surface levels of FGFR1 and the appearance of FGFR1 in the perinuclear ER/nuclear envelope (Fig. 1J).

All these data indicate that N-glycans of FGFR1 promote the progression of the receptor through the secretory pathway, thereby facilitating its accumulation in the plasma membrane. In the absence of N-glycans, autoactivated FGFR1 preferentially accumulates in the ER and in the nuclear envelope.

### N-glycans of the D2 and D3 domains are critical for targeting FGFR1 to the plasma membrane

**Fig. 2:**
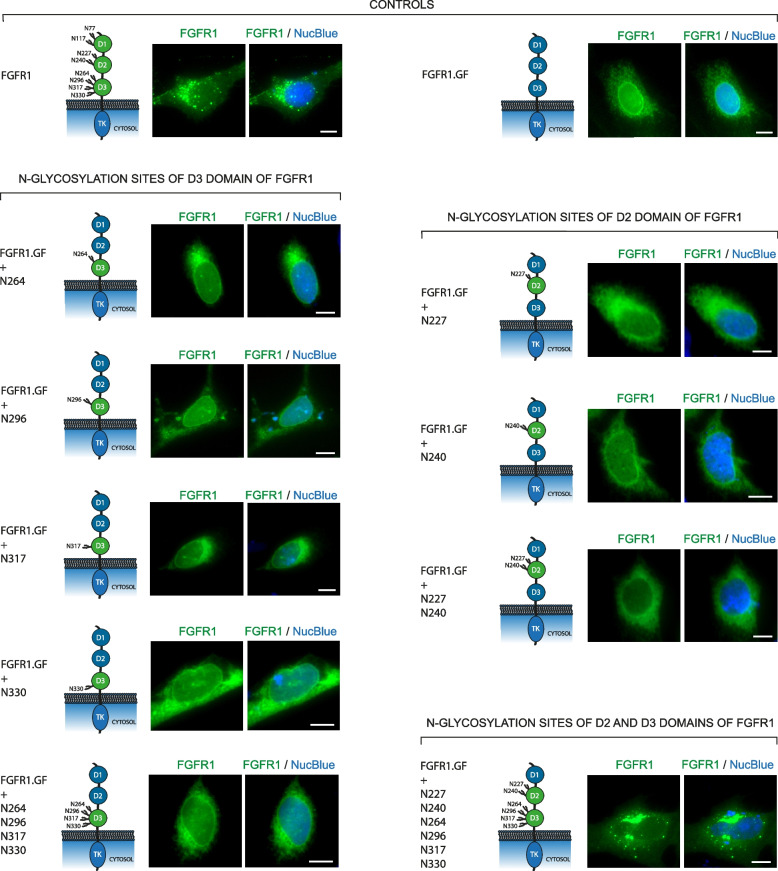
The N-glycosylation motifs of the D2 and D3 domain are critical for targeting of FGFR1 to the cell surface. Subcellular localization of distinct FGFR1 variants (the wild type FGFR1, FGFR1.GF and FGFR1.GF with reintroduced N-glycosylation sites) produced in U2OS cells. Schematic representations of the N-glycosylation status of the tested mutants are shown. Scale bar represent 10 µm

We then assessed which N-glycans of FGFR1 promote receptor trafficking to the cell surface by reintroducing N-glycosylation sites to FGFR1.GF and monitoring the subcellular localization of the receptor with fluorescence microscopy. We initially focused on reintroducing single N-glycosylation sites only into the D2 (two sites) and D3 (four sites) domains of FGFR1.GF, as FGFR1β, the receptor isoform lacking the D1 domain (containing two glycosylation sites) is efficiently directed to the cell surface and responds to externally added FGFs [54–57]. FGFR1.GF variants with any of the single N-glycosylation sites restored within the D3 domain (N264, N296, N317 or N330) were still localized to the nuclear envelope of U2OS cells (Fig. 2). Simultaneous restoration of all four glycosylation motifs within the D3 (N264/N296/N317/N330) was also not sufficient to direct FGFR1 into the secretory pathway, as this FGFR1 variant was still localized primarily to the nuclear envelope (Fig. 2).

Similarly, reintroduction of the N240 glycosylation motif present in the D2 domain could not restore FGFR1 trafficking to the cell surface (Fig. 2). In contrast, restoration of the N227 site of the D2 domain resulted in deprivation of the receptor from the nuclear envelope and its accumulation in the ER/Golgi (Fig. 2). Simultaneous reintroduction of both N-glycosylation sites of the D2 domain (N227 and N240) to FGFR1.GF prevented receptor accumulation in the nuclear envelope, and the receptor signal was mainly detected in the ER/Golgi, similar to the single N227 mutant (Fig. 2). Only the return of all six N-glycosylation sites in the D2 and D3 domains to FGFR1.GF resulted in the efficient transport of the receptor to the cell surface and the appearance of intracellular spots representing endosomes/secretory vesicles, similar to the wild type, fully N-glycosylated FGFR1 (Fig. 2).

These data implicate that N-glycans attached to N227 of the D2 domain of FGFR1 preclude accumulation of the receptor in the nuclear envelope, while extensive N-glycosylation in the D2 and D3 domains promote the progression of FGFR1 in the secretory route towards the plasma membrane.

### Nuclear envelope-localized FGFR1.GF interacts with a specific set of nuclear proteins

Although FGFR1 is an integral membrane protein containing the TM region, several previous studies have suggested the presence of soluble FGFR1 in the nuclear lumen [15, 16, 20, 21, 24–31, 33]. In contrast to these findings, our data clearly demonstrate that glycosylation-deficient FGFR1 mainly localizes to the nuclear envelope, implicating that after co-translational insertion into the ER membrane, non-glycosylated FGFR1 remains embedded in the membrane and diffuses laterally across the continuous ER/nuclear membranes to eventually accumulate in the nuclear envelope.

**Fig. 3:**
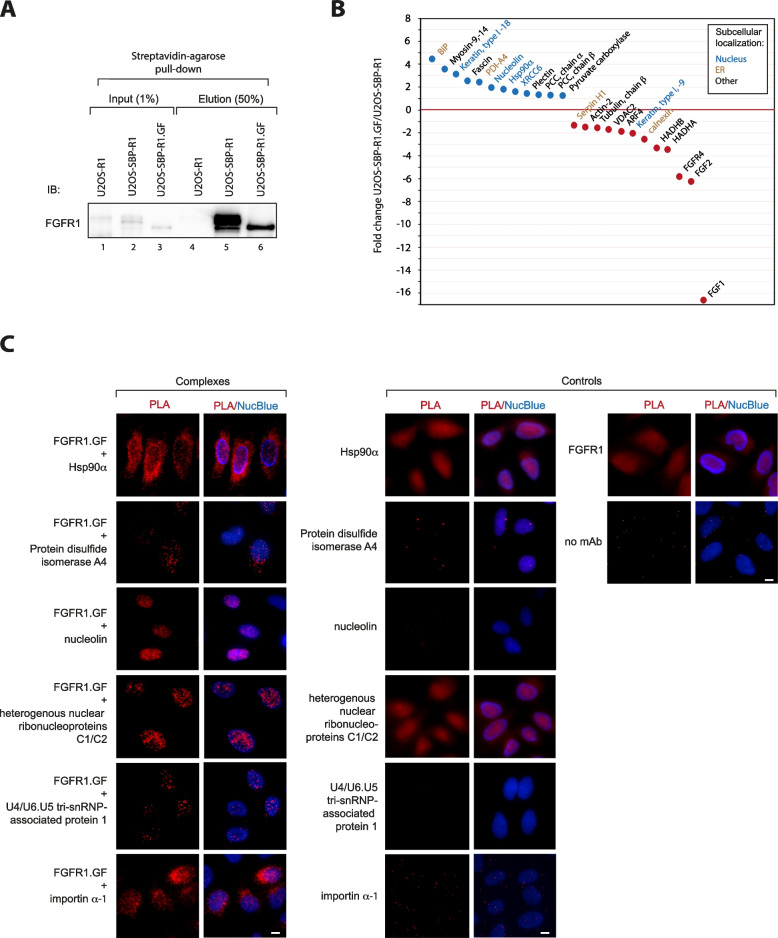
N-glycosylation shapes the interactome of FGFR1. **A**. Western blotting analysis of SBP-FGFR1 and SBP-FGFR1.GF affinity purification for mass spectrometry experiments. **B**. Quantitative MS-assessed selected proteins differentially interacting with SBP-FGFR1.GF and SBP-FGFR1 (full list available in Table S1). Nuclear-localized proteins are marked in blue, ER/Golgi proteins are marked in brown. Proteins for whose SBP-FGFR1.GF/SBP-FGFR1 ratio was around 1 or proteins with a q value above 0.05 are listed in Table S1 only. **C**. PLA confirmation of the interaction between FGFR1.GF and selected ER/nuclear proteins identified in MS experiments. Scale bar represent 10 µm

FGFR1 localized to the nuclear lumen has been shown to interact with a number of proteins involved in the regulation of gene expression [15, 16, 20, 21, 23–31, 33, 58]. To shed some light on the possible function of nuclear envelope-localized FGFR1, we identified the FGFR1.GF interactome using quantitative comparative mass spectrometry. We used U2OS-SBP-R1 and U2OS-SBP-R1.GF cells for affinity purification of the receptor variants and their interaction partners. As a purity control, we used U2OS-R1 cells stably producing non-tagged FGFR1. After efficient isolation of SBP-FGFR1.GF and SBP-FGFR1 (Fig. 3A), we performed quantitative label-free proteomics to identify proteins differentially interacting with plasma membrane-localized wild type SBP-FGFR1 and SBP-FGFR1.GF present in the nuclear envelope.

We detected dehydrogenase/reductase SDR family member 2, a mitochondrial and nuclear enzyme co-purifying only with SBP-FGFR1.GF (Table S1) [59]. We identified a set of intracellular proteins that were significantly enriched in SBP-FGFR1.GF relative to SBP-FGFR1, which include several proteins localized at least partially to the ER or nucleus, such as nucleolin, Hsp90α, BiP, protein disulfide isomerase A4 or X-ray repair cross-complementing protein 6 (Fig. 3B, Table S1). We also found several other nuclear proteins enriched in SBP-FGFR1.GF over SBP-FGFR1, such as importin subunit α-1, heterogeneous nuclear ribonucleoprotein C1/C2, U4/U6.U5 tri-snRNP-associated protein 1, but with a lower q-value (Table S1). In agreement with the predominant localization of FGFR1.GF to the nuclear envelope, co-purification of endogenous FGF1, FGF2, FGFR4 and glypican-1 with SBP-FGFR1.GF was dramatically reduced compared to the cell surface-localized wild type SBP-FGFR1 (Fig. 3B, Table S1).

We employed a proximity ligation assay (PLA) to confirm the MS-detected interactors of the nuclear envelope-localized FGFR1.GF. We detected strong PLA signals for FGFR1.GF-Hsp90α and FGFR1-protein disulfide isomerase A4 pairs in the perinuclear region, indicating their interaction in the ER (Fig. 3C). PLA signals were detected predominantly in the nucleus for FGFR1.GF complexes with nucleolin, heterogeneous nuclear ribonucleoprotein C1/C2, U4/U6.U5 tri-snRNP-associated protein 1 and importin subunit α-1 (Fig. 3C). In agreement with MS data, the interaction of the newly identified ER/nuclear binding partners of FGFR1.GF with wild type FGFR1 was minimal (Figure S3).

Consistent with the altered subcellular localization of FGFR1.GF, our MS and PLA data suggest that FGFR1.GF is spatially restricted from interacting with canonical components of FGF/FGFR1 signaling units, such as proteoglycans or secreted FGFs. Relocation of the glycosylation-deficient variant of FGFR1 to the nuclear envelope engages the receptor in a discrete network of interactions with several specific nuclear proteins involved in nuclear transport and mRNA processing.

### Impaired secretion of N-glycosylation-deficient FGFR1, rather than its compromised stability, drives receptor trafficking to the nuclear envelope

**Fig. 4:**
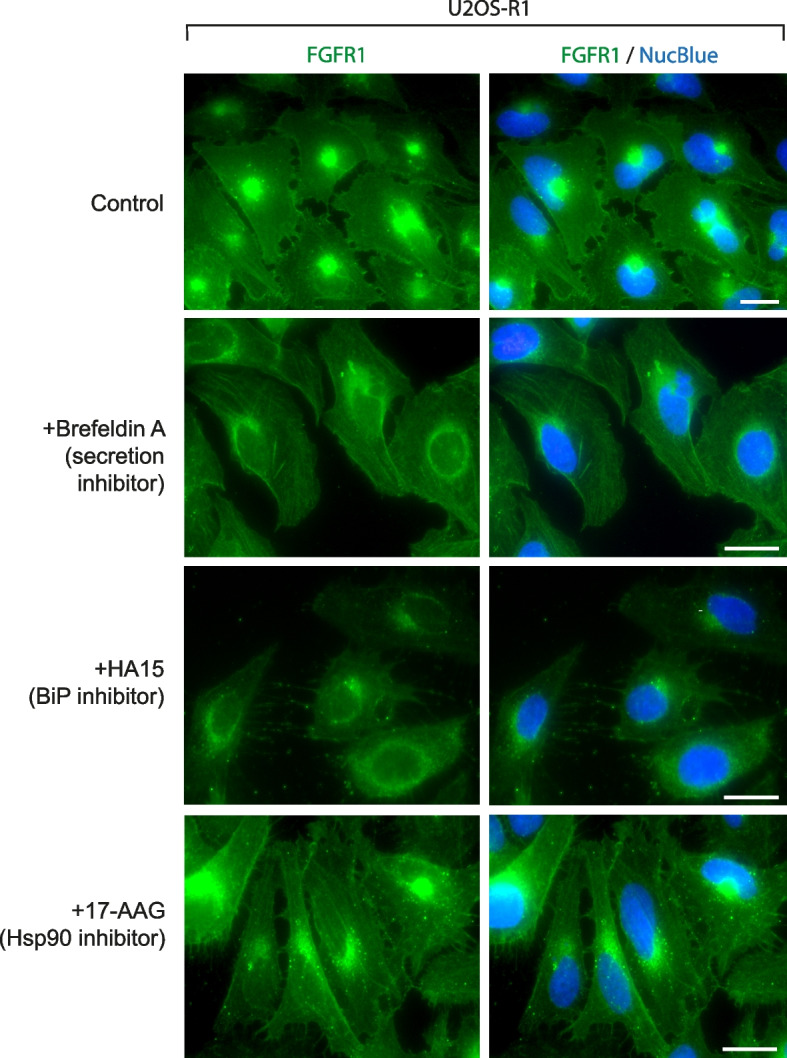
Blocked FGFR1 secretion facilitates its accumulation in the nuclear envelope. U2OS-R1 cells were treated with brefeldin A (secretion inhibitor), HA15 (BiP inhibitor) and 17-AAG (Hsp90 inhibitor) and FGFR1 localization was assessed with immunofluorescence (*n* = 3). Scale bar represents 20 µm

Since in MS and PLA experiments we observed largely enhanced interaction of FGFR1.GF with ER proteins involved in protein folding and quality control we wondered whether the re-direction of FGFR1.GF to the nuclear envelope is a result of FGFR1.GF destabilization or its blocked secretion. We treated U2OS-R1 cells with the protein secretion inhibitor brefeldin A and studied FGFR1 localization by fluorescence microscopy. As shown in Fig. 4, we observed in some cells sharp perinuclear signal of FGFR1, indicating FGFR1 accumulation in the ER/nuclear envelope. Treatment of U2OS-R1 cells with the BiP inhibitor HA15 caused a clearcut effect on FGFR1 subcellular localization with the appearance of perinuclear FGFR1 staining (Fig. 4). In contrast to the FGFR1.GF and FGFR1 signal, in U2OS-R1 cells treated with tunicamycin or brefeldin A, the perinuclear FGFR1 staining in U2OS-R1 cells in the presence of BiP inhibitor was much less sharp, indicating a predominant localization of FGFR1 to the ER (Fig. 4). Treatment of U2OS-R1 cells with the Hsp90 inhibitor 17-AAG had minimal impact on FGFR1 localization, seen as an appearance of numerous FGFR1-positive spots, largely absent in control cells (Fig. 4).

All these data indicate that N-glycosylation of FGFR1 predominantly drives the export of the receptor from the ER to the plasma membrane and that the absence of FGFR1.GF secretion, rather than receptor destabilization, causes re-location of the receptor to the nuclear envelope.

## Discussion

Although the nuclear localization of FGFR1 has been reported, the precise mechanism of nuclear translocation of the receptor is still unclear [15, 16, 25, 60]. It has been proposed that full length FGFR1 may reach the nucleus either by endocytosis of receptor molecules form the cell surface after FGF binding, or by the release of incompletely glycosylated soluble FGFR1 from the ER/Golgi before it reaches the cell surface [25, 35]. Noteworthy, in most cases FGFR1 was observed inside the nucleus, suggesting that the soluble form of FGFR1 is translocated into the lumen of the organelle [15]. In both scenarios, the involvement of importins and NPC in nuclear transport of FGFR1 was implicated [25].

**Fig. 5:**
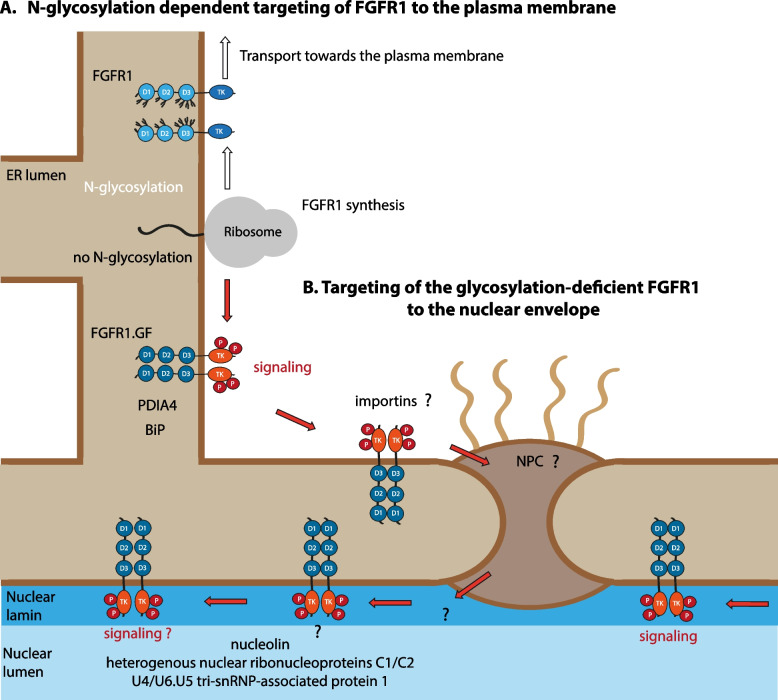
Hypothetical model of the N-glycosylation-dependent cellular trafficking of FGFR1. **A**. After co-translational synthesis in the ER, the wild type FGFR1 is N-glycosylated at several positions. The N227 site precludes FGFR1 transport to the nuclear envelope, while N-glycosylation sites in the D2 and D3 domain promote FGFR1 transport via the ER/Golgi/secretory vesicles to the plasma membrane, where the receptor becomes available for FGFs’ stimulation. During the transport to the cell surface, N-glycosylation of FGFR1 ensures low level of FGFR1 autoactivation in the intracellular compartments in the absence of FGFs. **B**. Signal peptide (SP)-driven co-translational ER targeting of the N-glycosylation-deficient FGFR1 (FGFR1.GF) results in the initial accumulation of FGFR1.GF in the ER, where it binds several protein folding and quality control factors, such as BiP or protein disulfide isomerase A4. In the absence of N-glycans, the extracellular region of FGFR1.GF undergoes unfolding and aggregation, initiating ligand-independent FGFR1.GF autoactivation. Alternatively, the absence of N-glycans in the properly folded extracellular region of FGFR1.GF facilitates FGFR1.GF dimerization and activation in the absence of FGFs. In both scenarios, intracellular FGFR1 displays a high degree of autoactivation. Lateral diffusion of the ER-trapped FGFR1.GF within the ER-membrane, which is continuous with the nuclear envelope, results in the transport of FGFR1.GF to the nuclear envelope. Importins and NPC are likely involved in this step. FGFR1.GF is retained in the nuclear envelope presumably by participating in complexes with a precise set of nuclear proteins. Importantly, FGFR1.GF localized to the nuclear envelope is highly kinase active, indicating the presence of a novel nuclear FGFR1 signaling cascade

Here, we identified N-glycosylation of FGFR1 as a targeting signal for the trafficking of the receptor to the cell surface and uncovered a novel route for the nuclear transport of FGFR1. In our model, N-glycosylation of FGFR1 functions as a switch defining the cellular localization of the receptor, where complete glycosylation of FGFR1 prevents its nuclear targeting and ensures efficient transport of the receptor to the plasma membrane (Fig. 5). Glycosylation-deficient mutant of FGFR1 does not reach the plasma membrane and localizes primarily to the nuclear envelope, implicating that it remains an integral membrane protein during nuclear targeting (Fig. 5). We hypothesize that FGFR1.GF is co-translationally inserted into the ER membrane by the ER translocon and diffuses laterally within ER membranes that are continuous with the nuclear envelope. Subsequently, we hypothesize that importins (which were previously implicated in the nuclear localization of FGFR1, and here detected as preferential interaction partners of FGFR1.GF) and NPC may promote accumulation of FGFR1 in the nuclear envelope (Fig. 5) [22, 61]. Although results of fluorescence microscopy experiments and MS/PLA interaction studies indicate the possible localization of FGFR1.GF to the inner leaflet of the nuclear envelope, insufficient resolution of applied imaging techniques and the lack of insights into the mechanism of FGFR1.GF putative relocation to the inner nuclear membrane makes this phenomenon hypothetical at this stage and clearly requires further studies.

Interestingly, not all of the eight N-glycosylation sites of the extracellular domain of FGFR1 are equally important for intracellular trafficking of FGFR1, as the N227 of the D2 domain appears critical for preventing FGFR1 accumulation in the nuclear envelope, while the N-glycans of the D2 and D3 domain of FGFR1 ensure the efficient transport of the receptor to the cell surface (Fig. 5). In agreement with our findings, Dunham-Ems et al*.,* observed a hypo-glycosylated pool the receptor in the nuclei of BAMC cells [34]. The FGFR2 C278F mutant associated with Cruzon craniosynostosis syndrome is characterized by diminished receptor N-glycosylation and nuclear localization of the receptor [62]. It is tempting to speculate that the N-glycosylation-dependent mechanism of FGFR1 cellular trafficking can be exploited by cells to rapidly and probably reversibly shape FGFR1 signaling under normal (e.g., changes in environmental conditions, cell differentiation) and pathological conditions, such as various malignancies [24, 60, 63, 64]. The physiological relevance of N-glycosylation-dependent trafficking of FGFR1clearly requires further investigation.

Nuclear FGFR1 has been shown to interact with the CBP/CREB complex, Nurr1, RNA polymerase II or FOXA1 and has so far been mainly associated with the regulation of gene expression in healthy and cancer cells [16]. We identified several novel binding partners of FGFR1.GF localized to the nuclear envelope and, consistent with previous functional implications for nuclear FGFR1, most of the newly identified proteins are involved in the regulation of gene expression. An open question is the role of the tyrosine kinase activity of nuclear FGFR1. The nuclear function of FGFR1 has so far been described as largely independent of the receptor tyrosine kinase [14, 35]. Since glycosylation-deficient FGFR1 and the nuclear FGFR2 C278F mutant display very high level of autoactivation, it is possible that there is an as yet uncharacterized FGFR1-dependent signaling pathway in the nucleus [62]. Most of the identified nuclear FGFR1 binding partners are intensively phosphorylated, but typically at serines and threonines, suggesting the involvement of other kinases acting between these nuclear proteins and FGFR1 in nuclear signaling cascades. Clearly, the role of tyrosine kinase activity in nuclear FGFR1 needs to be clarified.

The significance of FGFR1 N-glycosylation appears to be broader than previously anticipated. N-glycosylation of FGFR1 (at hitherto unidentified sites) modulates the interaction of the receptor with FGFs and proteoglycans [18, 65]. We have recently demonstrated that the N-glycans of FGFR1 provide binding sites for extracellular galectins that affect cellular transport and signaling of FGFR1 [19, 66]. Our data presented in this work and previous results of Hatch et al*.* on FGFR2 suggest that, in addition to their role in FGFR trafficking, N-glycans prevent FGFR autoactivation, possibly by sterically hindering interactions between FGFR monomers. Alternatively, the absence of N-glycans in the extracellular domain of FGFR1 causes unfolding and aggregation of the extracellular domain of FGFR1, which in turn brings close together TK domains on the other side of the ER/nuclear membrane, resulting in FGFR1 activation. This hypothesis is supported by interactions between FGFR1.GF and the ER-localized factors involved in protein folding, such as BiP, Hsp90α and protein disulfide isomerase A4.

In summary, our data implicate that the cell, by modifying the N-glycans of FGFR1, can adjust the cellular localization of the receptor (cell surface *vs* ER/Golgi *vs* nuclear envelope), alter FGFR1 signaling (ligand-dependent *vs* independent) and shape FGFR1 interactome. As the oncogenic activity of nuclear FGFR1, which so far has been reported to be kinase-independent, cannot be targeted with TKI or with FGFR1-specific antibodies/antibody-based therapeutics, expanding our knowledge of the function of nuclear FGFR1 and the mechanism of its nuclear trafficking may facilitate the development of novel cancer treatment strategies that inhibit the pool of previously untargeted nuclear FGFR1.

## Supplementary Information

**Additional file 1: Figure S1.** Analyses of FGFR1 activation by FGF1 in U2OS cell lines stably producing SBP-FGFR1and SBP-FGFR1.GF. **Figure S2.** Specificities of antibodies used in this study. A. **Figure S3.** PLA analysis of the interaction between FGFR1 variants and putative partner proteins in U2OS-SBP-R1 and U2OS-SBP-R1.GF

**Additional file 2.**

**Additional file 3: Table S1.** Label-free quantitative mass spectrometry analysis of proteins differentially interacting with SBP.FGFR1.GF and SBP-FGFR1.

## Data Availability

The datasets used in this study are available from the corresponding author on reasonable request.

## Abbreviations

AKT: Protein kinase B
BAMC: Bovine adrenal medullary cells
BiP: Binding immunoglobin protein
CBP: Histone acetyltransferase
CIE: Clathrin-independent endocytosis
CME: Clathrin-mediated endocytosis
COPB: Coatomer subunit beta
CREB: Cyclic AMP-responsive element-binding protein 1
D1-D3: Immunoglobulin-like domains
FGFR1.GF: N-glycosylation-deficient FGFR1
FGFRs: Fibroblast growth factor receptors
FGFs: Fibroblast growth factor factors
FHFs: Fibroblast growth factor homologous factors
FOX1A: Hepatocyte nuclear factor 3-alpha
LB: Lysis Buffer
MS: Mass spectrometry
mTOR: Mammalian target of rapamycin
NLS: Nuclear localization signal
NPC: Nuclear pore complex
Nurr1: Nuclear receptor subfamily 4 group A member 2
PI3K: Phosphoinositide 3-kinase
PLA: Proximity ligation assay
PLCγ: Phospholipase Cγ
SBP: Streptavidin binding peptide
SP: Signal peptide
STAT: Signal transducer and activator of transcription
TKI: Tyrosine kinase inhibitor
TM: Transmembrane region
WB: Washing Buffer
