# Supplementary material for: N-glycosylation acts as a switch for FGFR1 trafficking between the plasma membrane and nuclear envelope
Source: Cell Commun Signal. 2023 Jul 21;21:177. doi: 10.1186/s12964-023-01203-3 (PMC10362638; doi:10.1186/s12964-023-01203-3)
Supplement: Supplementary file 2 — Additional file 1: Figure S1. Analyses of FGFR1 activation by FGF1 in U2OS cell lines stably producing SBP-FGFR1and SBP-FGFR1.GF. Figure S2. Specificities of antibodies used in this study. A. Figure S3. PLA analysis of the interaction between FGFR1 variants and putative partner proteins in U2OS-SBP-R1 and U2OS-SBP-R1.GF [file 12964_2023_1203_MOESM1_ESM.pdf]

Supplementary Information #1 for:

N-glycosylation acts as a switch for FGFR1 trafficking between the plasma membrane and nuclear envelope

Paulina Gregorczyk[1#], Natalia Porębska[1#], Dominika Żukowska[1], Aleksandra Chorążewska[1], Aleksandra Gędaj[1], Agata Malinowska[2], Jacek Otlewski[1], Małgorzata Zakrzewska[1] and Łukasz Opaliński[1*]

[1]Faculty of Biotechnology, Department of Protein Engineering, University of Wroclaw, Joliot-Curie 14a, 50-383 Wroclaw, Poland

[2]Institute of Biochemistry and Biophysics, Polish Academy of Sciences, Pawińskiego 5a, 02-106 Warsaw, Poland

[#] These authors contributed equally to this work

*Correspondence should be addressed to L.O (lukasz.opalinski@uwr.edu.pl)

The file contains supplementary figures: Fig. S1, Fig. S2, Fig. S3 and corresponding figure legends.

Figure S1. Analyses of FGFR1 activation by FGF1 in U2OS cell lines stably producing SBP-FGFR1 (U2OS-SBP-R1) and SBP-FGFR1.GF (U2OS-SBP-R1). Cells were serum starved, treated with FGF1 (100 ng/mL, heparin 10U/mL), lysed and the level of phosphorylated FGFR1 (pFGFR), total FGFR1 and tubulin (loading control) was assessed with western blotting. The representative western blot from three independent experiments is shown (left panel). Middle panel: densitometric quantification of pFGFR signal (normalized to tubulin), displayed as a percentage of pFGFR level in FGF1-non treated samples (100%). Right panel: the densitometry-measured ratios of pFGFR/FGFR1 in U2OS-SBP-R1 and U2OS-SBP-R1.GF stable cell lines. Average values of 3 independent experiments (n=3) +/- SD are shown. Statistical analyses were performed with Student's t-test (*$p < 0.05$; **$p < 0.005$ and ***$p < 0.001$; n.s. – not significant).

Figure S2. Specificities of antibodies used in this study. A. FGFR1 staining with recombinant T-Fc antibody in FGFR1-negative parental U2OS cell line and in U2OS cells stably transfected with FGFR1 (U2OS-R1). Cells were incubated with 15 μg/mL of T-Fc for 30 min on ice, fixed, stained with NucBlue and Zenon-AF-488, and analyzed with fluorescence microscopy. Scale bars represent 20 μm. **B**. Serum-starved U2OS-R1 cells were pretreated with FGFR kinase inhibitor PD173074 (100 nM) for 15 min and incubated with FGF1 (100 ng/mL) for 30 min. Cells were either lysed and analyzed with western blotting with anti-pFGFR and FGFR1 antibodies (top panel) or fixed and analyzed with immunofluorescence using pFGFR antibody (bottom panel). Scale bars represent 20 μm.

Figure S3. PLA analysis of the interaction between FGFR1 variants and putative partner proteins in U2OS-SBP-R1 and U2OS-SBP-R1.GF. PLA confirmation of the interaction between FGFR1.GF and selected ER/nuclear proteins identified in MS experiments, and their differential interaction with the wild type N-glycosylated FGFR1 produced by U2OS-SBP-R1 cells. Scale bar represent 20 μm.

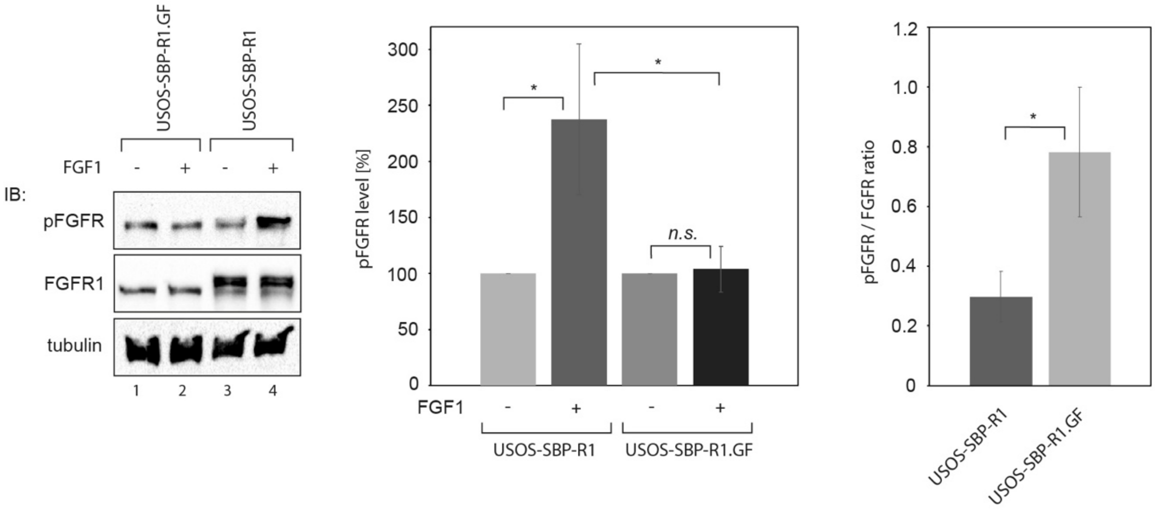
USOS-SBP-R1.GF
USOS-SBP-R1
FGF1
IB:
pFGFR
FGFR1
tubulin
1 2 3 4
pFGFR level [%]
n.s.
FGF1
USOS-SBP-R1
USOS-SBP-R1.GF
pFGFR / FGFR ratio
USOS-SBP-R1
USOS-SBP-R1.GF

Figure S1

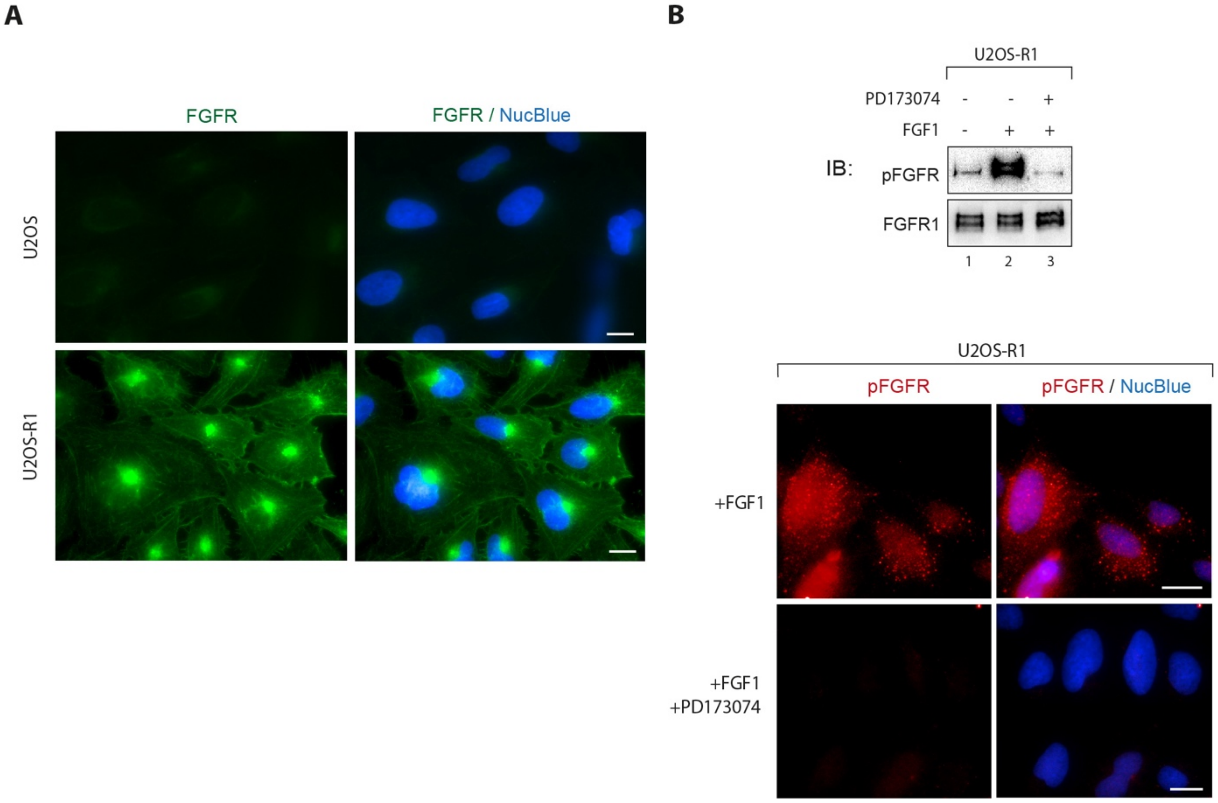
A
FGFR
FGFR / NucBlue
U2OS
U2OS-R1
B
U2OS-R1
PD173074 - - +
FGF1 - + +
IB:
pFGFR
FGFR1
1 2 3
U2OS-R1
pFGFR
pFGFR / NucBlue
+FGF1
+FGF1
+PD173074

Figure S2

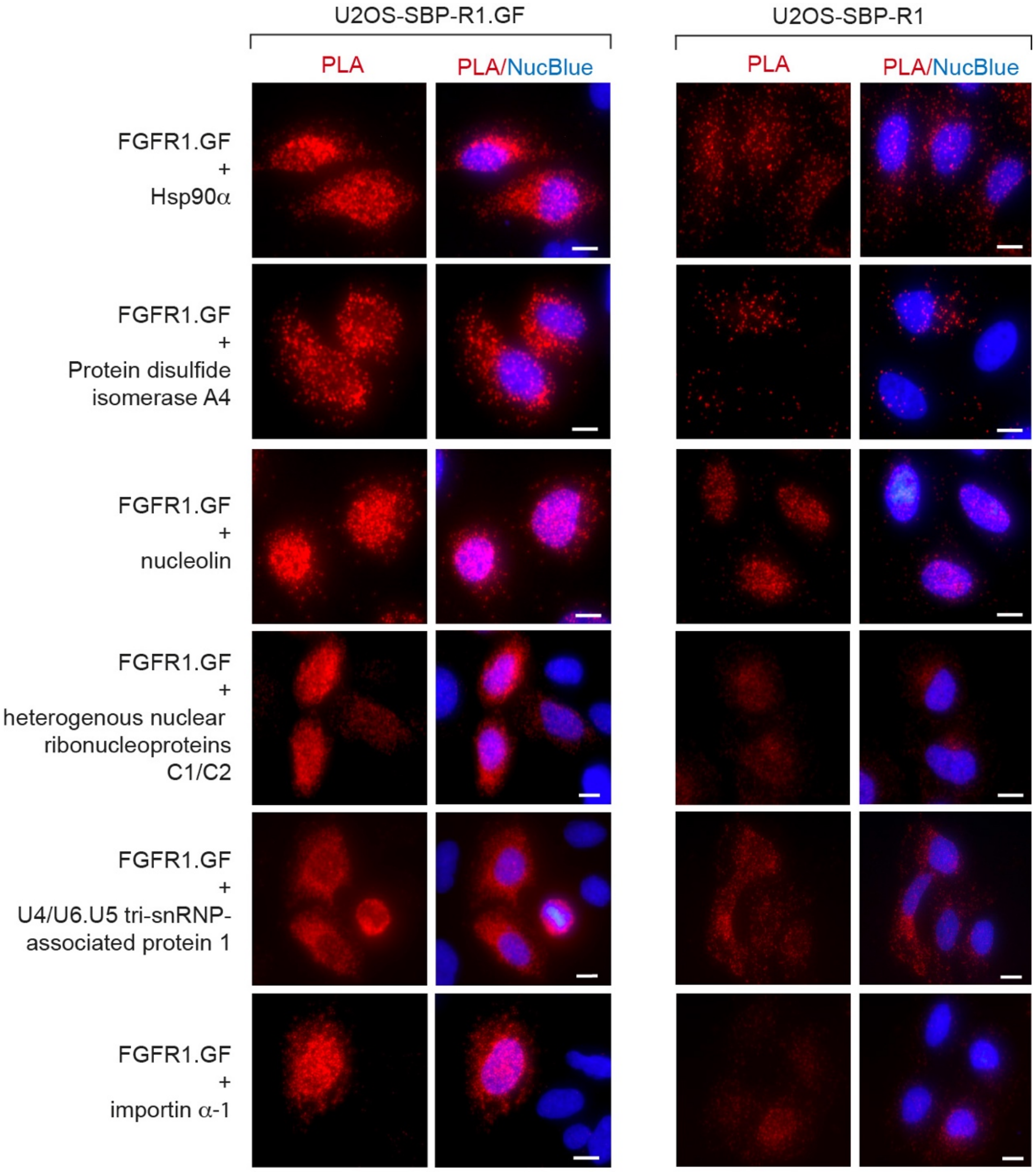
U2OS-SBP-R1.GF
U2OS-SBP-R1
PLA
PLA/NucBlue
PLA
PLA/NucBlue
FGFR1.GF + Hsp90α
FGFR1.GF + Protein disulfide isomerase A4
FGFR1.GF + nucleolin
FGFR1.GF + heterogenous nuclear ribonucleoproteins C1/C2
FGFR1.GF + U4/U6.U5 tri-snRNP-associated protein 1
FGFR1.GF + importin α-1

Figure S3
