# Supplementary material for: N-glycosylation acts as a switch for FGFR1 trafficking between the plasma membrane and nuclear envelope
Source: Cell Commun Signal. 2023 Jul 21;21:177. doi: 10.1186/s12964-023-01203-3 (PMC10362638; doi:10.1186/s12964-023-01203-3)
Supplement: Supplementary file 3 — Additional file 2. [file 12964_2023_1203_MOESM2_ESM.pdf]

Supplementary Information #2 for:

N-glycosylation acts as a switch for FGFR1 trafficking between the plasma membrane and nuclear envelope

Paulina Gregorczyk[1#], Natalia Porębska[1#], Dominika Żukowska[1], Aleksandra Chorążewska[1], Aleksandra Gędaj[1], Agata Malinowska[2], Jacek Otlewski[1], Małgorzata Zakrzewska[1] and Łukasz Opaliński[1]*

[1]Faculty of Biotechnology, Department of Protein Engineering, University of Wroclaw, Joliot-Curie 14a, 50-383 Wroclaw, Poland

[2]Institute of Biochemistry and Biophysics, Polish Academy of Sciences, Pawińskiego 5a, 02-106 Warsaw, Poland

[#] These authors contributed equally to this work

*Correspondence should be addressed to L.O (lukasz.opalinski@uwr.edu.pl)

The file contains original full-length blots presented in cropped versions in the Fig. 1 and Fig. 3, and Fig. S1

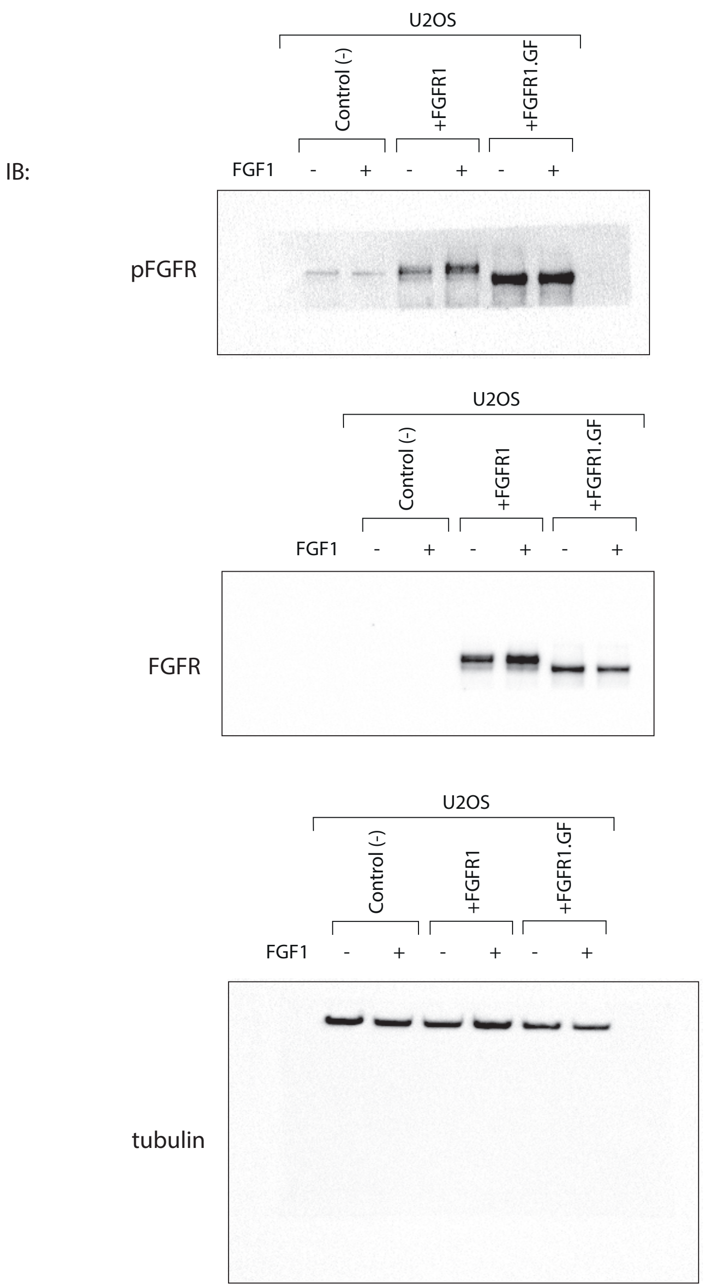

Uncropped blots from Fig. 1B

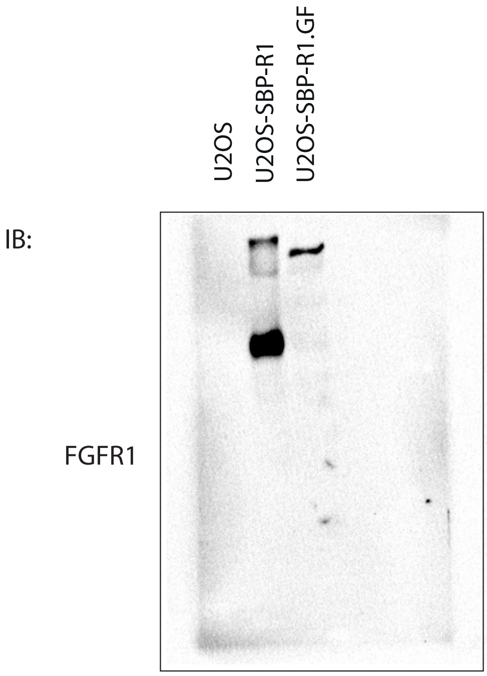

Uncropped blots from Fig. 1D

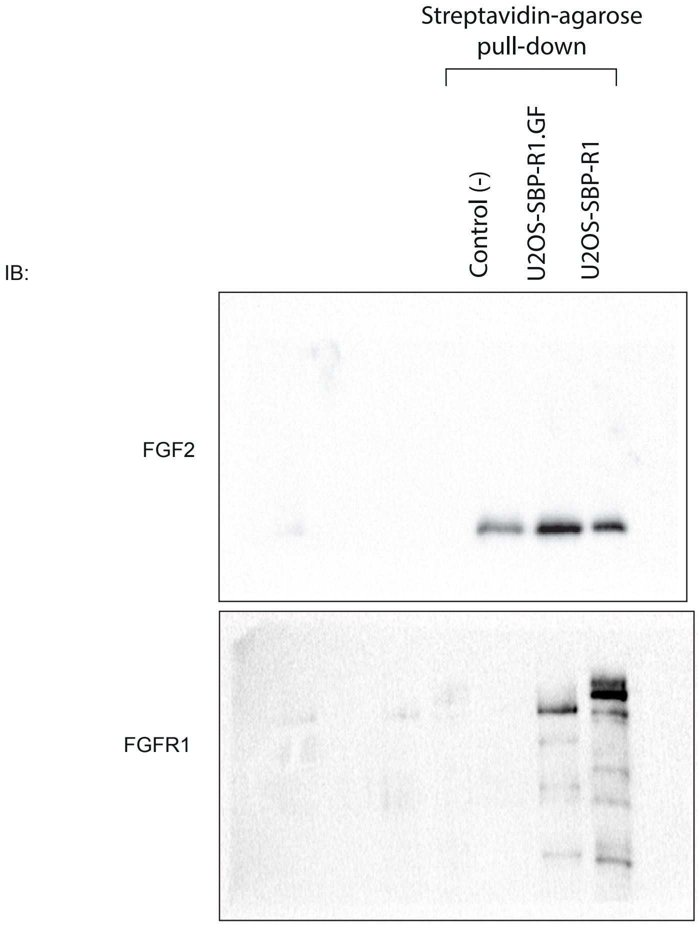

Uncropped blots from Fig. 1E

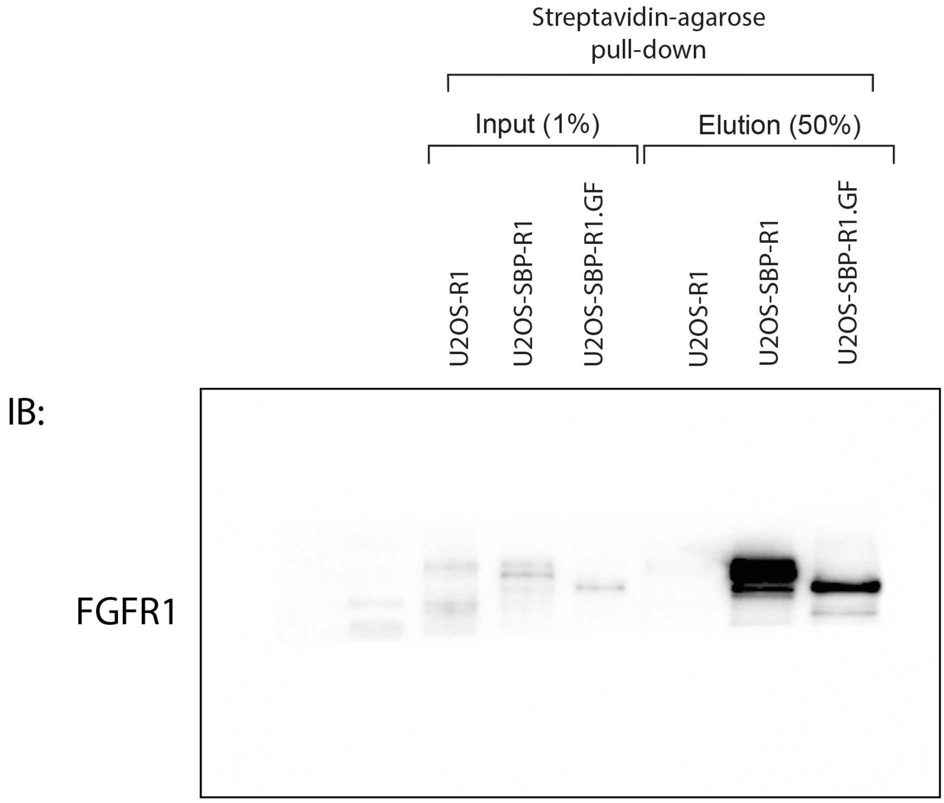

Uncropped blots from Fig. 3A

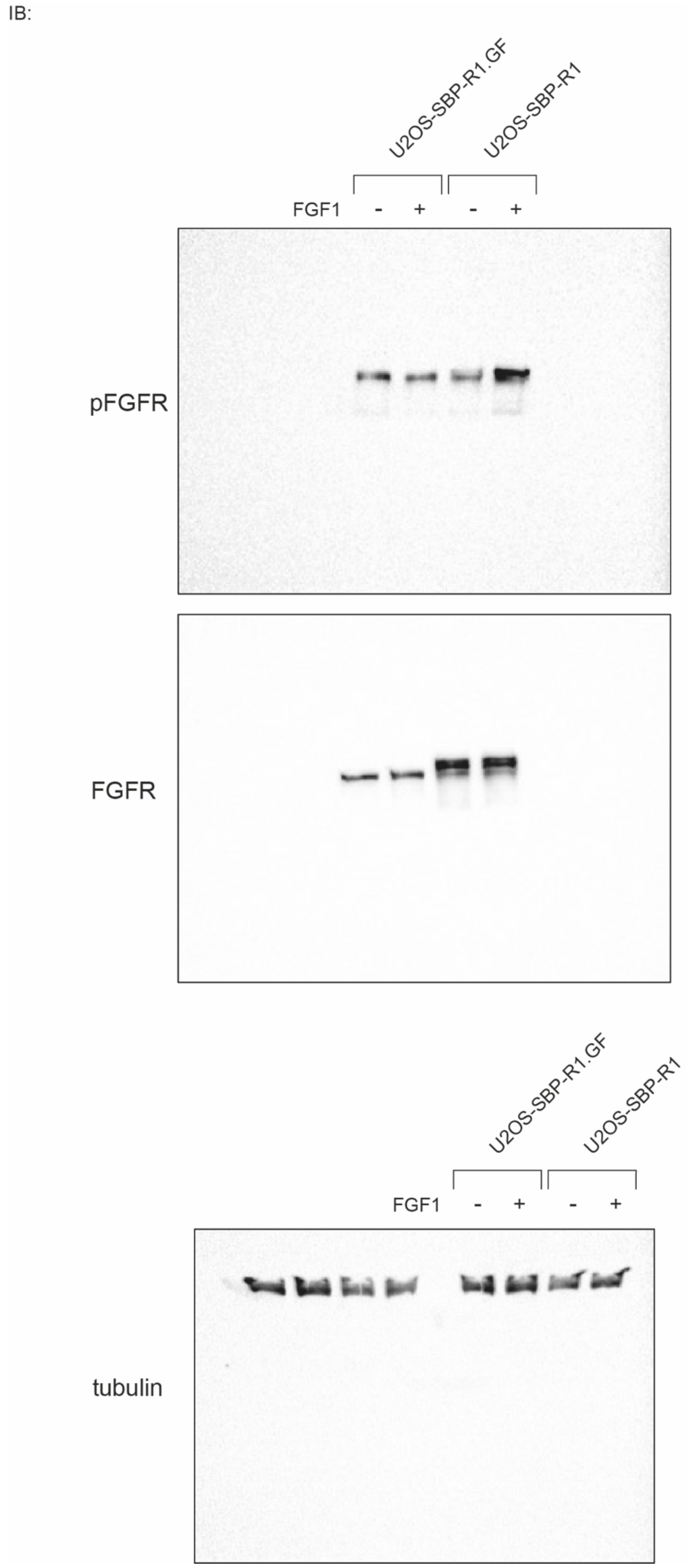

Uncropped blots from Fig. S1
